# Root cause analysis of hypoglycemic events in critically ill patients

**DOI:** 10.1186/cc12398

**Published:** 2013-03-19

**Authors:** A McDonald, J Krinsley

**Affiliations:** 1 Stamford Hospital, Stamford, CT, USA

## Introduction

Hypoglycemia (H) is a complication of intensive insulin (IN) therapy of critically ill patients and is independently associated with increased risk of mortality (M). Heightened attention to identified risk factors (RF) and causes of hypoglycemic events (HE) may lead to improvement in glycemic control. A limited literature describes RF for H but a detailed root cause analysis (RCA) of individual HE has not been published.

## Methods

This is a retrospective analysis of prospectively collected data including 835 patients admitted consecutively between 1 February 2012 and 31 October 2012 to the 16-bed medical-surgical ICU of a university-affiliated teaching hospital. The blood glucose (BG, mg/dl) target was 90 to 120 and the monitoring frequency was every 3 hours, or every 1 hour when the patient received i.v. IN infusion. HE data were collected by the bedside nurse, using a cutoff of BG <60, and information was abstracted from the unit's database. RF included: shock (SH), renal insufficiency (RI), hepatic failure (HF), mechanical ventilation (MV), and diabetes mellitus (DM). Attributable causes included: IN treatment, improper monitoring frequency (MF), interruption of feeding (FE), and spontaneous (SP). We measured rates of severe (<40) and mild (40 to 69) H, and compared them as well as percentage of values in the optimum range (70 to 139) and hyperglycemic range (>139) with data from the preceding 3 months (PRE).

## Results

Sixty-three (7.5%) patients sustained a total of 79 HE. They were older, more likely to be diabetic, and had higher APACHE IV predicted (37.3% vs. 15.4%, *P *0.0001) and observed (25.4% vs. 8.6%, *P *0.0001) M than those without H. RF: SH 24.1%; RI 24.1%; HF 8.9%; MV 46.8%; DM 36.5%. Attributable causes: IN 58.2%; MF 8.9%; FE 6.3%; SP 36.7%. BG <40, 40 to 69 and >139 decreased 78.6%, 39.0% and 9.3% (*P *= 0.0006, *P *0.0001, *P *0.0001). BG 70 to 139 increased 5.5% (*P *0.0001). In total, 4.2% and 22.4% of PRE patients sustained severe and mild H. In total, 0.7% and 14.1% of RCA patients sustained severe and mild H (*P *= 0.0029, *P *= 0.0003).

## Conclusion

IN treatment was associated with barely more than one-half of HE in this population of intensively monitored patients. Implementation of an initiative mandating RCA of every HE, including identification of RF and attributable causes, was associated with a significant improvement in glycemic control.

